# Comparative analysis of perinatal health outcomes among refugee subgroups and economic immigrants in Canada (2000–2017)

**DOI:** 10.1371/journal.pone.0321453

**Published:** 2025-04-29

**Authors:** Marwa Ramadan, Gabriel D. Shapiro, Seungmi Yang, Edward Ng, Bilkis Vissandjée, Zoua M. Vang

**Affiliations:** 1 Department of Sociology, McGill University, Montreal, Quebec, Canada; 2 Department of Community Medicine, Alexandria University, Alexandria, Egypt; 3 Department of Epidemiology, Biostatistics and Occupational Health, McGill University, Montreal, Quebec, Canada; 4 Health Analysis Division, Statistics Canada, Ottawa, Ontario, Canada; 5 School of Nursing, Public Health Research Centre (CReSP), Université de Montréal, SHERPA Research Institute, Montreal, Quebec, Canada; 6 School of Human Ecology, University of Wisconsin-Madison, Madison, Wisconsin, United States of America; Aarhus University: Aarhus Universitet, DENMARK

## Abstract

**Background:**

Refugees often face increased risks of poor perinatal health outcomes compared to native-born individuals and non-refugee immigrants. However, limited research has explored how birth outcomes vary across refugee subgroups in Canada, especially compared to economic immigrants and among refugee groups themselves. This study aimed to (1) compare the risk of preterm birth (PTB), small-for-gestational-age (SGA), large-for-gestational-age (LGA), stillbirth, and infant mortality between refugee subgroups and economic immigrants, and (2) examine differences among Government-Assisted Refugees (GARs), Privately Sponsored Refugees (PSRs), and In-Canada Refugees (ICRs).

**Methods:**

This population-based study used data from the Migrant Maternal and Infant Morbidity and Mortality (MIMMM) dataset, including 706,620 singleton births from 2000 to 2017. Generalized estimating equation models calculated adjusted risk ratios (aRRs) for birth outcomes, accounting for maternal and immigration-related factors.

**Results:**

All refugee subgroups had higher PTB (6.26–6.41 per 100 births) and LGA rates (8.65–9.17 per 100 births) but lower SGA rates (9.53–10.40 per 100 births) compared to economic immigrants (PTB: 5.95, LGA: 7.36, SGA: 10.96). After adjustment, GARs maintained higher PTB risks, and all refugee subgroups had lower SGA and higher LGA risks than economic immigrants. Within refugee subgroups, ICRs had higher SGA risks (aRR = 1.09; 95% CI: 1.04–1.14) than GARs, and PSRs (aRR = 1.22; 95% CI: 1.04–1.44) and ICRs (aRR = 1.28; 95% CI: 1.07–1.52) had higher stillbirth risks than GARs.

**Conclusion:**

Refugee women in Canada have higher risks of PTB and LGA births compared to economic immigrants. ICRs had higher risks of SGA births and stillbirths than other refugee subgroups but lower risks of SGA and stillbirths compared to economic immigrants. These disparities are partly explained by maternal and immigration-related factors. Further research is needed to better understand these factors and inform policies aimed at reducing health disparities among immigrant populations in Canada.

## Introduction

Globally, refugee women face increased risks of poor maternal and child health outcomes, including pregnancy complications, adverse birth outcomes, perinatal depression, and postnatal unmet health needs [1]. In the Global North—referring to high-income, industrialized countries primarily in North America, Western Europe, and parts of Oceania—, refugees face multiple barriers to healthcare access, including language barriers, cultural differences, structural racism, and low socioeconomic status [2]. Studies comparing perinatal health outcomes among immigrants and non-immigrant women show variability, with some finding no consistent differences [3–6], while others suggesting higher rates of adverse outcomes—such as preterm birth, perinatal mortality, and stillbirth—among refugees compared to non-refugee immigrants [1,7,8]. These findings underscore the importance of examining contextual factors, including admission categories, healthcare access, and social determinants of health, when analyzing health outcomes among displaced populations.

A refugee is defined by the 1951 Refugee Convention as a person who is unable or unwilling to return to their home country due to a well-founded fear of persecution based on race, religion, nationality, political opinion, or membership in a particular social group [9]. In Canada, refugees were recognized as a distinct class of immigrants following the Immigration Act of 1976 [10] and are generally classified into four main categories: Government-Assisted Refugees (GARs), who receive government-funded resettlement and support in collaboration with the UNHCR; Privately Sponsored Refugees (PSRs), introduced in 1979 to support the Indochinese crisis through private sponsorship networks [11,12]; In-Canada Refugees (ICRs), who apply for protection while already in Canada through a process formalized with the Immigration and Refugee Board under the Immigration Act of 1976 [13] and the Blended Visa Office-Referred (BVOR) Program, introduced in 2013 to address the Syrian refugee crisis, where the government and private sponsors jointly support refugees identified by the UNHCR ([14,15].

Refugee admission categories may play an important role in access to resources and social support, which can affect integration, healthcare access and perinatal health outcomes. In Canada, healthcare is delivered through a publicly funded system, offering universal coverage to citizens and permanent residents [16], but access to healthcare and support services for refugees varies by admission category. Although GARs and PSRs have the same eligibility for provincial health insurance, GARs typically receive one year of government-funded financial and settlement support, whereas PSRs primarily rely on social support from their private sponsors [17]. In contrast, ICRs often lack structured support systems and may encounter greater barriers to accessing healthcare and social services. A study of refugees in Ontario (2002–2017) found that PSRs were more likely to receive adequate prenatal care compared to GARs (69.3% vs. 62.3%) [18]. Another study found that asylum seekers tend to start antenatal care later and receive less adequate care than GARs and PSRs due to limited connections, financial barriers, and reduced health insurance coverage [17].

While most studies on refugee perinatal health in Canada compare refugees to native-born populations or non-refugee immigrants [6,19,20], recent research underscores the importance of examining differences within refugee groups [18]. A recent analysis of 7,980,650 births in Canada found that infants born to immigrants had higher risks of preterm birth (PTB), small-for-gestational-age (SGA) birth, and stillbirth compared to infants of Canadian-born parents, but lower risks of large-for-gestational-age (LGA) birth and neonatal death. Within immigrant groups, infants born to refugees collectively faced higher risks of early preterm birth and large-for-gestational-age (LGA) birth, but lower risks of small-for-gestational-age (SGA) birth compared to infants of economic-class immigrants. However, there were no significant differences in stillbirth, neonatal death, or overall infant mortality between refugee and economic [7]. Building on these findings, the present study disaggregates refugee perinatal health outcomes by admission category—GARs, PSRs, and ICRs—to achieve the following aims: (1) to compare perinatal health outcomes of these refugee subgroups to those of economic immigrants, a high human capital group known for strong health outcomes [21], and (2) to examine differences in adverse perinatal outcomes among the refugee subgroups themselves. This detailed categorization would provide critical insights into how different refugee resettlement pathways impact perinatal health outcomes, offering evidence to inform targeted interventions and policy development.

## Materials and methods

### Study design

We conducted an observational cross-sectional population-based study using linked vital statistics and administrative data to examine the perinatal outcomes of economic immigrants and refugees who officially landed in Canada from1980 onward and gave birth between 2000 and 2017.

### Data sources and study population

We utilized the Migrant Maternal and Infant Morbidity and Mortality (MMIMM) dataset [22] a unique data linkage conducted by Statistics Canada, to investigate perinatal outcomes among refugee subgroups and economic immigrants in Canada. The MMIMM dataset encompasses linked national-level data from vital statistics, hospital discharge records, and immigration landing records from the Longitudinal Immigration Database (IMDB) for all women with at least one live birth and/or stillbirth in Canada between 1993 and 2017. Data access and analysis was conducted at the research data center of McGill university from mid-February 2022 to mid-June 2024.

**Fig 1:**
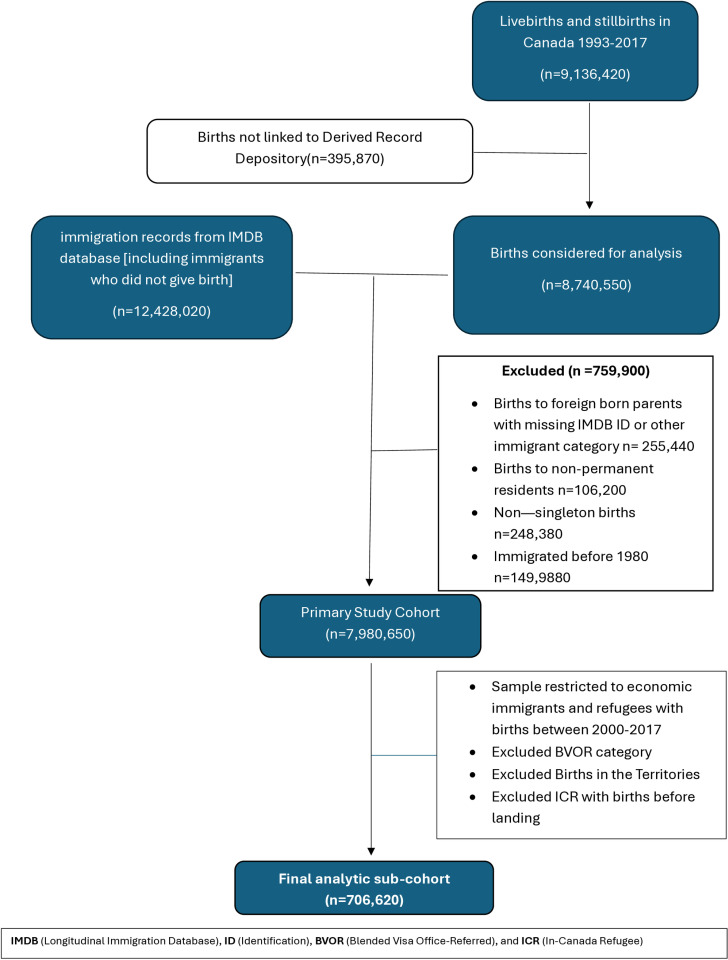
Flow diagram of the analytic sample selection. Adapted from: Yang S, Shapiro GD, Ng E, Vissandjée B, Vang ZM. (2024). Birth and postnatal outcomes among infants of immigrant parents of different admission categories and parents born in Canada: a population-based retrospective study. CMAJ, 196(12), E394–E409. https://doi.org/10.1503/cmaj.230878.

Building on the MMIMM data used by Yang et al [7]., our study population included a sub-cohort of economic immigrants and refugees who landed in Canada from 1980 onwards and who experienced at least one singleton birth between the years of 2000 and 2017 after official landing. Births to immigrant women in the territories were excluded from the analysis due to the small number of refugee women residing in these regions. The birth year restriction (2000–2017) was implemented due to documented data inconsistencies in the birth registration records of Ontario — the most populous province in Canada — during the early to mid-1990s, including errors in birth weight and gestational age, and challenges in linking live births to infant deaths from this period [23]. Missing data from the analytic sample were handled as follows: for variables with less than 5% missing observations, cases were excluded listwise from the analysis. For variables with between 5% and 20% missing data, we coded the missing values as a separate category to retain these observations in the analysis. There were no variables with more than 20% of observations missing in the dataset. Fig 1 shows the flow chart of the analytic sample selection.

### Measures

#### Dependent variables.

We investigated five perinatal outcomes: PTB, SGA birth, LGA birth, stillbirth, and infant mortality rate (IMR). PTBs is defined as a live birth occurring before 37 completed weeks of pregnancy[24]. SGA birth is defined as a birth weight below the 10^th^ percentile of a reference population by sex and gestational age, while LGA birth refers to babies with birth weights above the 90^th^ percentile of birth weight for babies of the same sex and gestational age, based on Canadian growth curves [25]. Stillbirth in all provinces except Quebec is defined as fetal death occurring at a minimum birth weight of 500 g or a gestational age of at least 20 weeks. In Quebec, stillbirth includes any fetal death at a weight over 500 g, regardless of gestational age. IMR is defined as the number of infant deaths within the first year of life per 1,000 live births [26]. Data for PTB, SGA, and LGA were derived from birth records, while mortality data were obtained from the Canadian Vital Statistics – Stillbirths and Deaths records.

#### Explanatory variables.

The primary independent variable in our study is the immigration category, as recorded in the IMDB database. We recategorized this variable into four groups: GARs, which include refugees and their dependents selected abroad by designated organizations based on a well-founded fear of persecution or risk of harm, who receive financial and settlement support from the Canadian government; PSRs, which include refugees and their dependents selected abroad and referred by private sponsors who provide financial and settlement assistance; ICRs, which include refugees and their dependents who claim asylum after arriving in Canada and await a decision on their refugee status, with the analysis in this group limited to birth outcomes after their refugee status was approved or granted; and Economic Immigrants, which include individuals and their dependents selected based on their potential economic contributions to Canada, including labor market needs, business ownership, investments, or provincial/territorial requirements. We did not include the BVOR category for the analysis since it was only introduced in 2013, and we did not have enough observations for meaningful comparisons.

#### Control variables.

We adjusted for the following child and maternal characteristics: child’s sex, mother’s age at delivery, parity, marital status at birth, year of birth, and province of residence. Immigration characteristics included the year of official landing, maternal age at landing (defined as the mother’s age at the time of officially entering Canada under her designated immigration status), the duration of residence in Canada at the time of the child’s birth, and the country and/or world region of origin. Based on the year of official landing in Canada, the immigration cohort was divided into four categories: 1980–1989, 1990–1999, 2000–2009, and 2010 or later. Maternal educational attainment at the time of landing, proficiency in French/English at the time of landing and whether social assistance was received in the year of the child’s birth were used as socioeconomic controls. Observations where the total family income reported during the infant’s birth year included social assistance income were coded as “1” and no social assistance as “0”.

### Statistical analyses

We first conducted a descriptive analysis of maternal and immigration characteristics, along with the crude rates of adverse perinatal outcomes among refugee subgroups and economic immigrants. Next, we performed both crude and adjusted regression analyses to estimate the risk ratios (RRs) of perinatal outcomes across the three refugee subgroups using generalized estimating equations (GEE) models with a log-link function and an exchangeable correlation structure. GEE models were selected to account for the potential correlation between multiple births within the same mother. These models were sequentially adjusted for maternal, child, immigration, and socioeconomic characteristics to account for potential confounding factors. Model 1 was adjusted for child and maternal characteristics (parity, child sex, year of birth, maternal age at delivery, marital status at birth, and province of residence). Model 2 further adjusted for immigration characteristics (world region of origin, immigrant cohort, maternal age at landing, and duration of residence in Canada). Model 3 (the fully adjusted model) accounted for maternal education at the time of landing, knowledge of an official language at landing, and receiving social assistance in the infant’s year of birth in addition to all variables from Model 2. This stepwise adjustment enabled us to assess the incremental cofounding impact of different groups of variables.

Two sets of analyses were performed. The first set compared the three refugee groups to economic immigrants, an immigrant group with documented health advantages [21]. The second set compared PSRs and ICRs to GARs to examine potential subgroup differences within the refugee population. GARs were selected as the reference group based on the assumption that, as government-supported refugees, they would have more favorable perinatal outcomes due to structured resettlement support, including financial assistance and facilitated access to healthcare services. This made them an appropriate baseline for comparison with PSRs and ICRs, who experience different levels of support upon arrival.

### Ethical approval

This study was based on the secondary analysis of restricted microdata that is only accessible through the Canadian Research Data Centers of Statistics Canada. Therefore, the study was exempt from the review process by the research ethics board of McGill University, and the need for informed consent was waived.

## Results

The analytic sample included 706,620 singleton births, of which 75% were to mothers who were economic immigrants, while 25% were to refugee women. Within the refugees, 42% of births occurred among ICRs, 35% among GARs, and 23% among PSRs. Table 1 provides an overview of maternal and immigration characteristics of the four groups.

**Table 1:** Child and maternal characteristics by refugee type and in comparison with economic immigrants, singleton births, 2000-2017.

	Economic Immigrants	Government Assisted Refugees (GARs)	Privately Sponsored Refugees (PSRs)	In Canada Refugees (ICRs)
	N (%)	N (%)	N (%)	N (%)
Total	528530	62680	42000	73110
**Parity**				
0	221670 (42.0)	21710 (34.6)	15790 (37.6)	26100 (35.7)
1	204220 (38.7)	18830 (30.0)	13950 (33.2)	23800 (32.6)
2+	102120 (19.3)	22140 (35.3)	12260 (29.2)	23220 (31.8)
**Child sex**				
Male	272420 (51.6)	32240 (51.4)	21360 (50.9)	37290 (51.0)
Female	255620 (48.4)	30430 (48.5)	20630 (49.1)	35810 (49.0)
**Year of birth**				
2000	16720 (3.2)	2540 (4.1)	2260 (5.4)	2600 (3.6)
2001	17880 (3.4)	2660 (4.2)	2120 (5.0)	2690 (3.7)
2002	19670 (3.7)	2600 (4.1)	2080 (5.0)	2810 (3.8)
2003	20500 (3.9)	2760 (4.4)	1990 (4.7)	2890 (4.0)
2004	22490 (4.3)	2760 (4.4)	1920 (4.6)	3180 (4.3)
2005	23720 (4.5)	3010 (4.8)	2060 (4.9)	3610 (4.9)
2006	25830 (4.9)	3220 (5.1)	1980 (4.7)	3980 (5.4)
2007	27860 (5.3)	3390 (5.4)	2190 (5.2)	4420 (6.0)
2008	28650 (5.4)	3520 (5.6)	2190 (5.2)	4530 (6.2)
2009	28320 (5.4)	3570 (5.7)	2180 (5.2)	4170 (5.7)
2010	30400 (5.8)	3550 (5.7)	2300 (5.5)	4450 (6.1)
2011	32210 (6.1)	3790 (6.0)	2440 (5.8)	4490 (6.1)
2012	35540 (6.7)	3920 (6.3)	2580 (6.1)	4580 (6.3)
2013	36030 (6.8)	3860 (6.2)	2540 (6.0)	4850 (6.6)
2014	37090 (7.0)	3890 (6.2)	2490 (5.9)	4490 (6.1)
2015	40510 (7.7)	4140 (6.6)	2680 (6.4)	5140 (7.0)
2016	43270 (8.2)	4530 (7.2)	2930 (7.0)	5200 (7.1)
2017	41310 (7.8)	4950 (7.9)	3040 (7.2)	5050 (6.9)
**Maternal characteristics**				
**Maternal Age at birth- groups**				
<20	2770 (0.5)	1870 (3.0)	520 (1.2)	1210 (1.7)
20-24	23540 (4.5)	9600 (15.3)	3990 (9.5)	8620 (11.8)
25-29	108760 (20.6)	18210 (29.1)	11380 (27.1)	21350 (29.2)
30-34	212440 (40.2)	19480 (31.1)	14930 (35.5)	23720 (32.4)
34-39	143980 (27.3)	10770 (17.2)	8920 (21.2)	14240 (19.5)
40+	36530 (6.9)	2760 (4.4)	2260 (5.4)	3970 (5.4)
Maternal Age at birth-average [years]	32.9 (SD: 4.94)	30.4 (SD: 5.43)	29.6 (SD: 5.09)	28.8 (SD: 5.05)
**Marital Status at birth**				
Single	39740 (7.5)	11220 (17.9)	6100 (14.5)	14400 (19.7)
Married	459530 (87.0)	43730 (69.8)	31210 (74.3)	50830 (69.5)
Widowed/Divorced/Separated	4690 (0.9)	1060 (1.7)	560 (1.3)	1420 (1.9)
Missing	24070 (4.6)	6670 (10.6)	4140 (9.9)	6460 (8.8)
**Province of residence**				
Atlantic	5600 (1.1)	1030 (1.6)	180 (0.4)	180 (0.2)
Quebec	110610 (20.9)	9990 (15.9)	5170 (12.3)	17770 (24.3)
Ontario	238140 (45.1)	28010 (44.7)	22970 (54.7)	45960 (62.9)
British Columbia	78360 (14.8)	7350 (11.7)	3960 (9.4)	3250 (4.4)
Other	95320 (18.1)	16270 (26.0)	9720 (23.1)	5940 (8.1)
**World Region of Origin**				
South and Central America, Caribbean	41920 (7.9)	10360 (16.5)	3600 (8.6)	12640 (17.3)
West Europe	54760 (10.4)	290 (0.5)	260 (0.6)	350 (0.5)
East Europe	56020 (10.6)	12340 (19.7)	7700 (18.3)	5630 (7.7)
Sub-Saharan Africa	28110 (5.3)	11020 (17.6)	8750 (20.8)	20390 (27.9)
Mideast/West Asia/ North Africa	75790 (14.4)	16850 (26.9)	10990 (26.2)	9900 (13.5)
South Asia	80570 (15.3)	8240 (13.1)	6910 (16.5)	19200 (26.3)
Southeast Asia	79080 (15.0)	2880 (4.6)	2960 (7)	330 (0.5)
East Asia	103350 (19.6)	470 (0.7)	390 (0.9)	4340 (5.9)
USA, Oceania & Other	8430 (1.6)	220 (0.4)	440 (1)	330 (0.5)
**Year of landing**				
1980-1989	54870 (10.4)	17150 (27.4)	12080 (28.8)	30 (0.04)
1990-1999	131690 (24.9)	17130 (27.3)	16530 (39.4)	26140 (35.8)
2000-2009	227360 (43.1)	22250 (35.5)	9200 (21.9)	38600 (52.8)
2010 +	114090 (21.6)	6140 (9.8)	4180 (10.0)	8340 (11.4)
**Maternal age at landing (Groups)**				
<5	15380 (2.9)	6680 (10.7)	4030 (9.6)	580 (0.8)
5-12	61050 (11.6)	14300 (22.8)	11370 (27.1)	6960 (9.5)
13-17	46910 (8.9)	9390 (15.0)	6020 (14.3)	10180 (13.9)
18+	404690 (76.6)	32300 (51.5)	20590 (49.0)	55390 (75.8)
Average age at landing (SD)	19.25 (5.38)	15.79 (7.14)	15.47 (7.09)	19.59 (4.62)
**Duration of residence in Canada**				
<2 years	82900 (15.7)	5830 (9.3)	2540 (6.0)	10060 (13.8)
2-5 years	184580 (35.0)	11770 (18.8)	5700 (13.6)	21340 (29.2)
6-10 years	110560 (20.9)	12630 (20.1)	7420 (17.7)	21200 (29.0)
11+ years	149980 (28.4)	32430 (51.7)	26360 (62.8)	20500 (28.0)
**Maternal education at landing**				
Less than high school diploma	185930 (35.2)	55620 (88.7)	36480 (86.9)	48120 (65.8)
High school diploma	110970 (21)	5210 (8.3)	4400 (10.5)	17750 (24.3)
Bachelor’s	171460 (32.5)	1730 (2.8)	1030 (2.5)	6370 (8.7)
Master’s+	59650 (11.3)	110 (0.2)	100 (0.2)	890 (1.2)
**Knowledge of official languages at landing**				
English only	287750 (54.5)	9430 (15.0)	10250 (24.4)	41460 (56.7)
French only	35620 (6.7)	2630 (4.2)	1220 (2.9)	11180 (15.3)
English and French	67840 (12.8)	1070 (1.7)	670 (1.6)	3310 (4.5)
Neither English nor French	136820 (25.9)	49540 (79)	29860 (71.1)	17180 (23.5)
**Received family social assistance payments in delivery year**				
Yes^a^	44550 (8.4)	21560 (34.4)	5810 (13.8)	20560 (28.1)

^a^analyzed as a binomial variable: “0” for receiving no social assistance (reference category) and “1” for receiving social assistance.

* value for Pearson’s Chi-Square Test of Independence

Economic immigrants in our study cohort were more likely to be married (87%) compared to the three refugee subgroups (ranging from 70% to 75%). All refugee subgroups had a substantially higher proportion of higher parity (two or more previous births) than economic immigrants (ranging from 29% to 35% among refugees versus 19% among economic migrants) and giving birth under the age of 20 was more common among refugees (Table 1). Economic immigrants predominantly originated from countries in Asia and the Middle East, while refugee women mainly originated from countries in the Middle East, Sub-Saharan Africa, Eastern Europe, and South Asia. More than 75% of GARs and PSRs resided in Ontario and the western provinces (excluding British Columbia), where large settlement communities exist. Similarly, the majority of economic immigrants and ICRs lived in Ontario (63%), followed by Quebec (24%), which are the two most populous provinces in Canada. ICR and economic immigrants tended to be older at landing, with more than 75% being over 18 years of age at landing, compared to half of GAR and PSR. More than 50% of GAR and PSR had been living in Canada for over 10 years at the time of giving birth in our study cohort, compared to 28% of economic immigrants and ICR. At time of landing, more than 85% of GAR and PSR had less than a high school diploma and more than 70% did not speak English or French. In addition, GAR and ICR were slightly more likely to receive social assistance in the delivery year compared to other sub-groups (8% economic immigrants, 34% GAR, 14% PSR, and 28% ICR).

**Fig 2:**
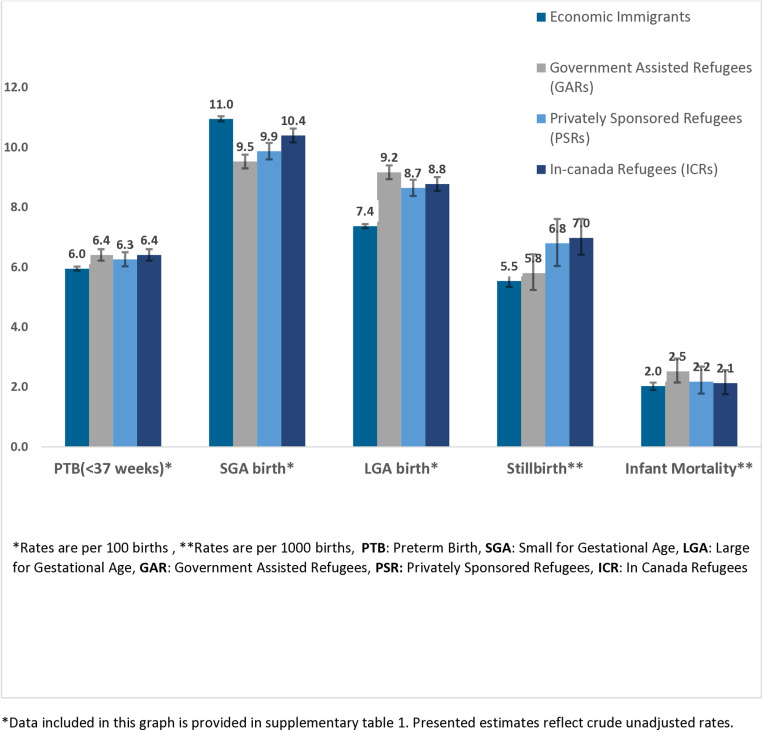
Crude perinatal outcome rates (95% CI) for refugees and economic immigrants, singleton births, 2000-2017.

Fig 2 (S1 Table for numerical results) shows the crude perinatal outcome rates for singletons born between 2000 and 2017 by refugee subgroup and in comparison, with economic immigrants. Additionally, S1 Table includes the crude perinatal outcome rates for the Canadian-born population during the same period, serving as an additional reference group for immigrant categories. Results from the adjusted analysis comparing the refugee subgroups to economic immigrants are shown in Table 2.

**Table 2:** Risk ratios (95% CI) for adverse perinatal outcomes by refugee type and in comparison, with economic class immigrants, singleton births, 2000-2017.

Outcome	Government Assisted Refugees (GARs)				Privately Sponsored Refugees (PSRs)				In-Canada Refugees (ICRs)			
	Crude	Adjusted			Crude	Adjusted			Crude	Adjusted		
		Model 1	Model 2	Model 3		Model 1	Model 2	Model 3	Model 1	Model 2	Model 3	
	RR (95% CI)	aRR (95% CI)	aRR (95% CI)	aRR (95% CI)	RR (95% CI)	aRR (95% CI)	aRR (95% CI)	aRR (95% CI)	RR (95% CI)	aRR (95% CI)	aRR (95% CI)	aRR (95% CI)
**Preterm birth (<37 weeks)**	1.08 (1.04,1.11)	1.10 (1.06,1.14)	1.09 (1.05,1.13)	1.06 (1.02,1.1)	1.06 (1.02,1.1)	1.06 (1.01,1.1)	1.03 (0.98,1.07)	1.01 (0.96,1.06)	1.08 (1.04,1.11)	1.13 (1.09,1.17)	1.10 (1.06,1.14)	1.07 (1.03,1.1)
**SGA birth**	0.87 (0.84,0.89)	0.88 (0.86,0.91)	0.93 (0.91,0.96)	0.91 (0.88,0.94)	0.90 (0.87,0.93)	0.90 (0.87,0.93)	0.92 (0.89,0.95)	0.90 (0.87,0.94)	0.94 (0.92,0.97)	0.99 (0.96,1.01)	0.91 (0.89,0.94)	0.89 (0.87,0.91)
**LGA birth**	1.26 (1.22,1.3)	1.23 (1.19,1.27)	1.13 (1.09,1.16)	1.12 (1.08,1.16)	1.19 (1.14,1.23)	1.15 (1.11,1.19)	1.07 (1.03,1.11)	1.05 (1.01,1.1)	1.20 (1.17,1.24)	1.14 (1.11,1.18)	1.15 (1.12,1.19)	1.14 (1.11,1.18)
**Stillbirth**	1.05 (0.94,1.17)	0.69 (0.61,0.77)	0.64 (0.57,0.73)	0.66 (0.57,0.75)	1.22 (1.08,1.38)	0.85 (0.75,0.96)	0.79 (0.69,0.9)	0.79 (0.69,0.91)	1.26 (1.15,1.38)	0.95 (0.86,1.04)	0.79 (0.71,0.88)	0.79 (0.71,0.88)
**Infant Mortality**	1.24 (1.04,1.47)	1.17 (0.98,1.4)	1.21 (1.0,1.46)	1.15 (0.94,1.42)	1.07 (0.86,1.33)	1.04 (0.84,1.30)	1.04 (0.83,1.31)	1.02 (0.81,1.3)	1.06 (0.89,1.25)	1.12 (0.94,1.33)	1.07 (0.89,1.3)	1.03 (0.85,1.25)

In our analysis, all three refugee’ sub-groups (GARs, PSRs, and ICRs) had higher crude rates of PTB than economic immigrants (Fig 2, S1 Table). After adjusting for child, maternal, immigration, and socioeconomic characteristics (Model 3), o statistically significant associations remained only for GARs (aRR= 1.06, 95% Confidence Interval (CI): 1.02, 1.10) and ICRs (aRR= 1.07, 95% CI: 1.03, 1.10) who showed slightly higher PTB rates in fully adjusted analyses compared to economic immigrants (Table 2).

All three refugee subgroups had lower rates of SGA and higher rates of LGA compared to economic immigrants. Specifically, SGA rates ranged from 9.53 to 10.40 per 100 births within the sub-groups of refugees versus 10.96 per 100 births among economic immigrants. Additionally, LGA rates ranged from 8.65 to 9.17 per 100 births among refugees versus 7.37 per 100 births among economic immigrants (Fig 2, S1 Table). Controlling for child, maternal, immigration, and socioeconomic characteristics did not substantially diminish these group differences (Table 2). For example, compared to economic immigrants, the fully adjusted model for SGA showed aRR = 0.91 (95% CI=0.88, 0.94) among GARS; aRR = 0.90 (95% CI=0.87, 0.94) among PSRs; and aRR= 0.89 (95% CI=0.87, 0.91) among ICRs. Meanwhile, the fully adjusted model of LGA showed aRR = 1.12 (95% CI=1.08,1.16) among GARs; aRR = 1.05 (95% CI=1.01,1.10) among PSRs; and aRR=1.14 (95% CI=1.11, 1.18) among ICRs.

Reference category is economic immigrants. Model 1 was adjusted for child and maternal characteristics (parity, child sex, year of birth, maternal age, marital status, and province of residence). Model 2 additionally adjusted for immigration characteristics (world region of origin, immigrant cohort, and duration in Canada). Model 3 further adjusted for characteristics at landing for education and knowledge of official language as well as for receipt of social assistance at birthyear. All models adjusted for clustering of births by mother. SGA: Small for gestational age, LGA: large for gestational age.

In crude analysis, both PSRs and ICRs showed higher stillbirth rates compared to economic immigrants (Fig 2, S1 Table). However, after adjustments, the rates were lower for GARs (aRR = 0.66, 95% CI: 0.57, 0.75), PSR (aRR = 0.79, 95% CI: 0.69, 0.91), and ICRs (aRR = 0.79, 95% CI: 0.71, 0.88) in comparison to economic immigrants (Table 2). This reversal in the association was largely attributable to factors significantly linked to a higher risk of stillbirth in our analysis, including being single, maternal age over 35 years, first-time motherhood (nulliparity), a shorter duration of residence in Canada (less than 2 years), and the inability to speak both English and French. In contrast, no significant differences in infant mortality rates (IMR) were observed between the refugee subgroups and economic immigrants (Table 2). Notably, the crude stillbirth rates were lower, while the crude IMR was higher in the Canadian-born population compared to both economic immigrants and each refugee subgroup during the study period which may indicate a potential health selection bias (S1 Table).

Table 3 shows the crude and the adjusted risk ratios of adverse perinatal outcomes among refugee subgroups, with GARs serving as a reference category. There were no significant differences in PTB rates between GARs and the other refugee groups. ICRs had slightly higher SGA rates (aRR = 1.09, 95% CI: 1.04, 1.14) compared to GARs. Both PSRs (aRR = 0.93, 95% CI: 0.89, 0.97) and ICRs (aRR = 0.94, 95% CI: 0.89, 0.98) had lower LGA rates compared to GARs. Both PSRs (aRR = 1.22, 95% CI: 1.04,1.44) and ICRs (aRR = 1.28, 95% CI: 1.07, 1.52) exhibited higher stillbirth rates compared to GARs. Meanwhile, there were no significant differences in IMR risk ratios across refugee subgroups (Table 3).

**Table 3:** Risk ratios (95% CI) for adverse perinatal outcomes among Privately Sponsored Refugees (PSR) and In-Canada Refugees (ICR) compared to Government Assisted Refugees (GAR), singleton births, 2000-2017.

Outcome	Privately Sponsored Refugees (PSRs)				In-Canada Refugees (ICRs)			
	Crude	Adjusted			Crude	Adjusted		
		Model 1	Model 2	Model 3		Model 1	Model 2	Model 3
	RR (95% CI)	aRR (95% CI)	aRR (95% CI)	aRR (95% CI)	aRR (95% CI)	aRR (95% CI)	aRR (95% CI)	aRR (95% CI)
Preterm birth (<37 weeks)	0.98 (0.93,1.03)	0.97 (0.93,1.03)	0.95 (0.91,1.0)	0.96 (0.91,1.01)	1.00 (0.96,1.05)	1.01 (0.97,1.06)	1.01 (0.96,1.07)	1.00 (0.94,1.05)
SGA birth	1.04 (0.99,1.08)	1.04 (0.99,1.08)	1.00 (0.96,1.04)	1.01 (0.97,1.05)	1.09 (1.05,1.13)	1.14 (1.10,1.18)	1.04 (1.00,1.08)	1.09 (1.04,1.14)
LGA birth	0.94 (0.90,0.98)	0.92 (0.88,0.96)	0.93 (0.89,0.98)	0.93 (0.89,0.97)	0.96 (0.92,0.99)	0.91 (0.88,0.95)	0.96 (0.92,1.00)	0.94 (0.89,0.98)
Stillbirth	1.17 (1.00,1.37)	1.24 (1.06,1.45)	1.22 (1.04,1.44)	1.22 (1.04,1.44)	1.20 (1.05,1.37)	1.36 (1.18,1.56)	1.30 (1.12,1.52)	1.28 (1.07,1.52)
Infant Mortality	0.87 (0.67,1.12)	0.92 (0.70,1.20)	0.90 (0.68,1.17)	0.94 (0.71,1.24)	0.85 (0.68,1.07)	0.99 (0.79,1.25)	0.91 (0.70,1.18)	0.96 (0.71,1.28)

Reference category is Government Assisted Refugees (GAR). Model 1 was adjusted for child and maternal characteristics (parity, child sex, birth cohort, maternal age, marital status, and province of residence). Model 2 additionally adjusted for immigration characteristics (world region of origin, immigrant cohort, and duration in Canada). Model 3 further adjusted for characteristics at landing for education, and knowledge of official languages and receipt of social assistance at the year of birth. All models adjusted for clustering of births by mother. SGA: Small for gestational age, LGA: large for gestational age.

## Discussion

This study evaluated adverse perinatal health outcomes among refugee women in Canada, categorized by admission type. Building on our previous analysis comparing immigrant groups to the native-born population [7], we highlighted the importance of recognizing that immigrants in Canada are not a homogeneous group and that disaggregating health outcomes by immigrant category is essential. Our findings in this study revealed that refugees, regardless of their admission category, experienced different birth outcomes compared to economic immigrants. Specifically, PTB rates and LGA rates among refugee subgroups were generally higher compared to economic immigrants. Additionally, crude stillbirth rates were elevated among refugee subgroups compared to economic immigrants. However, this higher crude risk of stillbirth among refugees were largely explained by factors such as nulliparity, advanced maternal age (>35 years), single parenthood, short duration of residence in Canada (<2 years), and limited proficiency in English and French. Furthermore, our comparative analysis of refugee subgroups identified that ICRs are particularly vulnerable, experiencing higher risks of SGA and stillbirths compared to other refugee subgroups.

The high rates of PTB observed among refugee subgroups compared to economic immigrants align with previous studies in Canada which have reported increased odds of PTB among refugees relative to non-refugee women [6,27]. Several factors may explain these disparities in adverse birth outcomes, such as PTB and LGA births, between refugees and economic immigrants. Research in OECD countries, including Canada, indicates that refugees face numerous barriers to healthcare access—such as language difficulties, cultural differences, structural racism, and low socioeconomic status—which can hinder their ability to navigate the healthcare system effectively [2]. These challenges are compounded by the heightened stress refugees often experience during the resettlement process [28–31].The association is further supported by existing literature, which links maternal stress to an increased risk of premature labor particularly when stress occurs during early pregnancy [32]. In contrast, economic immigrants – who typically have better literacy, language skills, employment status, and social integration – have been shown to exhibit particularly robust health [21].

Although our crude analysis aligns with existing literature showing that refugees are more prone to stillbirths than economic immigrants or native-born populations [21],this association was reversed after adjusting for maternal characteristics in Model 1. Specifically, once factors such as maternal age, parity, and single parenthood were accounted for, the initially higher stillbirth rates among refugees compared to economic immigrants were no longer observed or even reversed. This suggests that maternal characteristics substantially explain the crude differences in stillbirth rates. Furthermore, in Models 2 and 3, after additional adjustment for immigration-related factors—such as duration of residence in Canada and language proficiency—the risk of stillbirth among refugees further decreased. This highlights the significant impact of integration-related challenges on perinatal outcomes. Barriers to resettlement, including a shorter duration of residence in Canada (less than two years) and limited proficiency in English and French, likely hinder timely access to healthcare and essential support services, contributing to adverse perinatal outcomes.

Although maternal age was included in our models, it is important to note that the mean maternal age was higher among economic immigrants compared to refugee subgroups. Advanced maternal age is a well-established risk factor for stillbirth and preterm birth (PTB). However, in our study, younger refugee women still experienced higher rates of PTB, suggesting that factors beyond maternal age—such as limited healthcare access, socioeconomic disadvantage, and chronic psychosocial stress—play a more substantial role in shaping adverse perinatal outcomes among refugee populations.

Our adjusted analysis showed that ICR had higher rates of SGA and stillbirths compared to GAR. The more precarious circumstances surrounding ICRs’ entry and settlement in Canada may contribute to their increased risk of adverse birth outcomes. Specifically, ICR are more likely to experience socioeconomic challenges, difficulties in accessing healthcare and/or low levels of social support compared to other refugee subgroups [1,33,34]. These findings are consistent with previous studies reporting that refugee claimants or asylum seekers were more likely to experience low birth weight and/or SGA compared to other refugee subgroups [35,36]. However, it is important to recognize that ICRs still had lower odds of SGA and stillbirths compared to economic immigrants and showed no significant differences in infant mortality. This nuance suggests that while ICRs face specific vulnerabilities relative to other refugee groups (GARs), their risk profiles differ when compared to economic immigrants. Additionally, our analysis focused exclusively on ICRs after their asylum claims were approved, potentially underestimating the full extent of their health vulnerabilities during the period before gaining official refugee status. Given these findings, further research is warranted to explore how pre- and post-migration factors uniquely affect ICRs’ perinatal health.

Our study has several limitations. First, the analysis was limited to births occurring after landing in Canada, which may underestimate vulnerabilities within the ICR subgroup. Some ICR women may have resided in Canada for years and given birth before officially obtaining refugee status. During this period, they likely faced restricted access to healthcare services, limited social support, and socioeconomic challenges, which could have placed them at higher risk for adverse perinatal outcomes. As a result, our findings may underrepresent the full extent of health risks experienced by ICR women. If this bias did not exist, the disparities in outcomes such as PTB, SGA, and stillbirth rates among ICRs may have been even more pronounced. Second, despite covering an 18-year period, the small number of stillbirths and infant deaths may have limited the precision of analyses for these rare outcomes. Third, our estimations of LGA and SGA were based on Canadian growth curves, which may not fully capture ethnic variations among immigrant groups. Additionally, we could not assess whether refugees were presented later for prenatal care, a factor known to impact gestational age estimation particularly if gestational age was instead based on self-reported last menstrual period rather than early ultrasound, affecting the reliability of SGA and LGA classifications [37]. Finally, due to dataset limitations, we lacked information on prior adverse pregnancy outcomes and pre-migration risk factors (e.g., duration in refugee camps), which may have contributed to the differences observed among refugee subgroups and economic immigrants. However, parity in our analysis was based on complete reproductive history, not just births occurring after arrival in Canada. We also adjusted for maternal age at landing to account for differences in age at arrival between groups. While these adjustments help mitigate some biases, unmeasured pre-migration factors may still influence both cases and controls, potentially contributing to the observed differences.

This study adds to the growing body of literature on refugee perinatal health in resettlement contexts by highlighting significant variations in birth outcomes among refugee women in Canada compared to economic migrants. To our knowledge, this is the first study to examine perinatal outcomes over an 18-year period while disaggregating results by refugee admission category. Our findings emphasize that refugees are not a homogeneous group and that disaggregating health outcomes by immigrant category is critical for understanding and addressing their distinct health challenges. Notably, the heightened risks of stillbirth and SGA births among ICRs compared to other refugee subgroups highlight the compounded vulnerabilities this group faces. However, it is important to note that ICRs still exhibited lower adjusted risk of SGA and stillbirths compared to economic immigrants. These findings suggests that while ICRs experience certain disadvantages relative to other refugee groups, their risk profile differs when compared to economic immigrants. Future research should further explore how pre-migration exposures—such as prolonged stays in transit countries, displacement in refugee camps, and exposure to conflict and trauma—interact with post-migration resettlement conditions, including barriers to healthcare access, language barriers, socioeconomic challenges, and limited social support. A deeper understanding of how these interconnected factors contribute to disparities in perinatal outcomes among refugee subgroups can guide the development of more effective, evidence-based policies aimed at reducing health inequities and improving maternal and infant health outcomes in immigrant populations.

## Conclusion

In this national study spanning 18 years, we found that refugee women in Canada, regardless of their admission category, experienced higher rates of PTB and LGA births compared to economic immigrants. Significant differences were also observed among refugee subgroups, with ICRs showing higher rates of SGA births and stillbirths compared to GARs and PSRs. Some of these disparities were largely explained by factors such as maternal age, nulliparity, single parenthood, shorter duration of residence in Canada, and limited proficiency in English and French. A deeper understanding of the pre- and post-migration factors contributing to these outcomes is essential for healthcare professionals to tailor antenatal care and reduce adverse perinatal outcomes in vulnerable refugee populations. Further research is needed to inform policies aimed at reducing health disparities among immigrant populations in Canada.

## Supporting information

S1 Table Perinatal outcomes for Canadian-born, refugees and economic immigrants, singleton live births and stillbirths, 2000–2017. (DOCX)

S2 Ethics exemption. (PDF)

S3 Analytic code. (DOCX)
