# Supplementary material for: Comparative analysis of perinatal health outcomes among refugee subgroups and economic immigrants in Canada (2000–2017)
Source: PLoS One. 2025 Apr 29;20(4):e0321453. doi: 10.1371/journal.pone.0321453 (PMC12040250; doi:10.1371/journal.pone.0321453)
Supplement: S1 Table — (DOCX) [file pone.0321453.s001.docx]

**Supplementary Tables**

S1 Table. Perinatal outcomes (Rates (95% CI), N) for Canadian-born, refugees and economic immigrants, singleton live births and stillbirths, 2000-2017

|  | Canadian Born | | Economic immigrants | | | Government Assisted Refugees (GARs) | | Privately Sponsored Refugees (PSRs) | | In-Canada Refugees (ICRs) | |
| --- | --- | --- | --- | --- | --- | --- | --- | --- | --- | --- | --- |
| N | **4459450** | | **525100** | | | **62310** | | **41720** | | **72600** | |
|  | Rates (95% CI) | N | | Rates (95% CI) | N | Rates (95% CI) | N | Rates (95% CI) | N | Rates (95% CI) | N |
| Preterm birth (<37 weeks) ^a^ | 6.19 (6.16,6.21) | 275740 | | 5.95 (5.89,6.02) | 31250 | 6.41 (6.22,6.60) | 4000 | 6.26 (6.03,6.50) | 2620 | 6.41 (6.23,6.59) | 4660 |
| SGA birth ^a^ | 7.59 (7.56,7.61) | 338770 | | 10.96 (10.88,11.05) | 57530 | 9.53 (9.30,9.76) | 5940 | 9.87 (9.60,10.15) | 4120 | 10.40 (10.18,10.63) | 7540 |
| LGA birth ^a^ | 11.8 (11.77,11.83) | 525150 | | 7.37 (7.30,7.44) | 38690 | 9.17 (8.94,9.40) | 5710 | 8.65 (8.38,8.92) | 3600 | 8.78 (8.58,8.99) | 6370 |
| Stillbirth ^b^ | 4.77 (4.7,4.83) | 21300 | | 5.54 (5.34,5.74) | 2920 | 5.80 (5.24,6.43) | 370 | 6.80 (6.04,7.61) | 280 | 6.98 (6.40,7.61) | 510 |
| Infant Mortality ^b^ | 3.02 (2.97,3.07) | 13420 | | 2.03 (1.9,2.15) | 1060 | 2.52 (2.15,2.95) | 150 | 2.18 (1.78,2.68) | 90 | 2.13 (1.82,2.50) | 160 |

^a^ Rates are per 100 births

^b^ Rates are per 1000 births
SGA: Small for gestational age, LGA: large for gestational age.
