# Supplementary material for: Comparative analysis of perinatal health outcomes among refugee subgroups and economic immigrants in Canada (2000–2017)
Source: PLoS One. 2025 Apr 29;20(4):e0321453. doi: 10.1371/journal.pone.0321453 (PMC12040250; doi:10.1371/journal.pone.0321453)
Supplement: S2 — (PDF) [file pone.0321453.s002.pdf]

THE UNIVERSITY of WISCONSIN MADISON

Minimal Risk Research IRB
3/4/2024

**Submission ID number:**	2024-0230
**Title:**	Mind the Gap: Disparities in Perinatal Health among Foreign-born Women in Canada
**Principal Investigator:**	Zoua M. Vang
**Point-of-contact:**	Zoua M. Vang
**IRB Staff Reviewer:**	Sherry Holcomb

The IRB determined that the proposed activity is not research involving human subjects as defined by DHHS and FDA regulations.

You have identified the following financial sources to support the activities in this IRB application:

None.

IRB review and approval by this organization is not required. This determination applies only to the activities described in the IRB submission and does not apply should any changes be made. If changes are made and there are questions about whether these activities are research involving human subjects, please submit a new request to the IRB for a determination.

ARROW will permanently delete this certified application in 3 years. If you need a copy of the materials associated with this application, please log into your ARROW account and use the "Export Applications to PDF" activity found under the 'Activities' section in the submission's worskspace. Also, be sure to save a copy of this notification which can be found under the correspondence tab in the submission's workspace.

If you have general questions, please contact the Minimal Risk Research IRB at 608-263-2362. For questions related to this submission, contact the assigned staff reviewer.
