# Supplementary material for: Comparative analysis of perinatal health outcomes among refugee subgroups and economic immigrants in Canada (2000–2017)
Source: PLoS One. 2025 Apr 29;20(4):e0321453. doi: 10.1371/journal.pone.0321453 (PMC12040250; doi:10.1371/journal.pone.0321453)
Supplement: S3 — (DOCX) [file pone.0321453.s003.docx]

**Analytic Code
____________________**

**Author: Marwa Ramadan
Date: 202204
Purpose: Analysis of perinatal outcomes among refugee subgroups and Immigrants**

**# Bookmark #1

Part 1: importing and merging variables of interest from IMDB tax files FROM 2009 (ONE YEAR BEFORE OUR BIRH COHORT) TO 2017

Keeping variables of interest from tax files

XTIRCF-'Total income (ISD),family',

MKINCF- 'Market Income,family' ,

EI___F- 'Employment inusrance benefits',

TRPINF- 'Government transfer payments,family',

SASPYF-'Social assistantce payments,family',

EINS_F-'Employment insurance benefits,family',

LIMXTI-'Low income status,before tax'**;

LIMATI- lOW INCOME STATUS AFTER TAX

NAIC- Main principal industry

NAIC2- secondary principal industry

NAICCI NUMBER OF DIFFERENCES

*/

/* please note from IMDB immigration database LM_INTENTION_CD Definition: Contains 11 skill levels based on the National Occupational

Classification. This variable refers to the intended skill level of the immigrant at

the time of admission.

Source: Immigrant Landing FilE

*/

* Update 20220408- expanding the birth cohort and merge with tax files from 1999 to 2016 - the following loop is to prepare tax files fom 1999 to 2008 to shorten do file

/*

forvalues i = 1999/2016 {

use "S:\IMDB_BDIM_AllYears\IMDB_BDIM_2020_v1\data_donnees\stata\core_imdb\imdb_t1ff_"`i'"_f3_v1.dta", clear

keep IMDB_ID XTIRCF MKINCF EI___F TRPINF SASPYF EINS_F LIMXTI LIMATI NAIC*

save "T:\Projet 6899\Marwa_wokingfolder\Tax_files\"`i'".dta", replace

}

*/

use "S:\IMDB_BDIM_AllYears\IMDB_BDIM_2020_v1\data_donnees\stata\core_imdb\imdb_t1ff_1999_f3_v1.dta", clear

keep IMDB_ID XTIRCF MKINCF EI___F TRPINF SASPYF EINS_F LIMXTI LIMATI NAIC*

save "T:\Projet 6899\Marwa_wokingfolder\Tax_files\1999.dta", replace

use "S:\IMDB_BDIM_AllYears\IMDB_BDIM_2020_v1\data_donnees\stata\core_imdb\imdb_t1ff_2000_f3_v1.dta", clear

keep IMDB_ID XTIRCF MKINCF EI___F TRPINF SASPYF EINS_F LIMXTI LIMATI NAIC*

save "T:\Projet 6899\Marwa_wokingfolder\Tax_files\2000.dta", replace

use "S:\IMDB_BDIM_AllYears\IMDB_BDIM_2020_v1\data_donnees\stata\core_imdb\imdb_t1ff_2001_f3_v1.dta", clear

keep IMDB_ID XTIRCF MKINCF EI___F TRPINF SASPYF EINS_F LIMXTI LIMATI NAIC*

save "T:\Projet 6899\Marwa_wokingfolder\Tax_files\2001.dta", replace

use "S:\IMDB_BDIM_AllYears\IMDB_BDIM_2020_v1\data_donnees\stata\core_imdb\imdb_t1ff_2002_f3_v1.dta", clear

keep IMDB_ID XTIRCF MKINCF EI___F TRPINF SASPYF EINS_F LIMXTI LIMATI NAIC*

save "T:\Projet 6899\Marwa_wokingfolder\Tax_files\2002.dta", replace

use "S:\IMDB_BDIM_AllYears\IMDB_BDIM_2020_v1\data_donnees\stata\core_imdb\imdb_t1ff_2003_f3_v1.dta", clear

keep IMDB_ID XTIRCF MKINCF EI___F TRPINF SASPYF EINS_F LIMXTI LIMATI NAIC*

save "T:\Projet 6899\Marwa_wokingfolder\Tax_files\2003.dta", replace

use "S:\IMDB_BDIM_AllYears\IMDB_BDIM_2020_v1\data_donnees\stata\core_imdb\imdb_t1ff_2004_f3_v1.dta", clear

keep IMDB_ID XTIRCF MKINCF EI___F TRPINF SASPYF EINS_F LIMXTI LIMATI NAIC*

save "T:\Projet 6899\Marwa_wokingfolder\Tax_files\2004.dta", replace

use "S:\IMDB_BDIM_AllYears\IMDB_BDIM_2020_v1\data_donnees\stata\core_imdb\imdb_t1ff_2005_f3_v1.dta", clear

keep IMDB_ID XTIRCF MKINCF EI___F TRPINF SASPYF EINS_F LIMXTI LIMATI NAIC*

save "T:\Projet 6899\Marwa_wokingfolder\Tax_files\2005.dta", replace

use "S:\IMDB_BDIM_AllYears\IMDB_BDIM_2020_v1\data_donnees\stata\core_imdb\imdb_t1ff_2006_f3_v1.dta", clear

keep IMDB_ID XTIRCF MKINCF EI___F TRPINF SASPYF EINS_F LIMXTI LIMATI NAIC*

save "T:\Projet 6899\Marwa_wokingfolder\Tax_files\2006.dta", replace

use "S:\IMDB_BDIM_AllYears\IMDB_BDIM_2020_v1\data_donnees\stata\core_imdb\imdb_t1ff_2007_f3_v1.dta", clear

keep IMDB_ID XTIRCF MKINCF EI___F TRPINF SASPYF EINS_F LIMXTI LIMATI NAIC*

save "T:\Projet 6899\Marwa_wokingfolder\Tax_files\2007.dta", replace

use "S:\IMDB_BDIM_AllYears\IMDB_BDIM_2020_v1\data_donnees\stata\core_imdb\imdb_t1ff_2008_f3_v1.dta", clear

keep IMDB_ID XTIRCF MKINCF EI___F TRPINF SASPYF EINS_F LIMXTI LIMATI NAIC*

save "T:\Projet 6899\Marwa_wokingfolder\Tax_files\2008.dta", replace

use "S:\IMDB_BDIM_AllYears\IMDB_BDIM_2020_v1\data_donnees\stata\core_imdb\imdb_t1ff_2009_f3_v1.dta", clear

keep IMDB_ID XTIRCF MKINCF EI___F TRPINF SASPYF EINS_F LIMXTI LIMATI NAIC*

save "T:\Projet 6899\Marwa_wokingfolder\Tax_files\2009.dta", replace

use "S:\IMDB_BDIM_AllYears\IMDB_BDIM_2020_v1\data_donnees\stata\core_imdb\imdb_t1ff_2010_f3_v1.dta", clear

keep IMDB_ID XTIRCF MKINCF EI___F TRPINF SASPYF EINS_F LIMXTI LIMATI NAIC*

save "T:\Projet 6899\Marwa_wokingfolder\Tax_files\2010.dta", replace

use "S:\IMDB_BDIM_AllYears\IMDB_BDIM_2020_v1\data_donnees\stata\core_imdb\imdb_t1ff_2011_f3_v1.dta", clear

keep IMDB_ID XTIRCF MKINCF EI___F TRPINF SASPYF EINS_F LIMXTI LIMATI NAIC*

save "T:\Projet 6899\Marwa_wokingfolder\Tax_files\2011.dta", replace

use "S:\IMDB_BDIM_AllYears\IMDB_BDIM_2020_v1\data_donnees\stata\core_imdb\imdb_t1ff_2012_f3_v1.dta", clear

keep IMDB_ID XTIRCF MKINCF EI___F TRPINF SASPYF EINS_F LIMXTI LIMATI NAIC*

save "T:\Projet 6899\Marwa_wokingfolder\Tax_files\2012.dta", replace

use "S:\IMDB_BDIM_AllYears\IMDB_BDIM_2020_v1\data_donnees\stata\core_imdb\imdb_t1ff_2013_f3_v1.dta", clear

keep IMDB_ID XTIRCF MKINCF EI___F TRPINF SASPYF EINS_F LIMXTI LIMATI NAIC*

save "T:\Projet 6899\Marwa_wokingfolder\Tax_files\2013.dta", replace

use "S:\IMDB_BDIM_AllYears\IMDB_BDIM_2020_v1\data_donnees\stata\core_imdb\imdb_t1ff_2014_f3_v1.dta", clear

keep IMDB_ID XTIRCF MKINCF EI___F TRPINF SASPYF EINS_F LIMXTI LIMATI NAIC*

save "T:\Projet 6899\Marwa_wokingfolder\Tax_files\2014.dta", replace

use "S:\IMDB_BDIM_AllYears\IMDB_BDIM_2020_v1\data_donnees\stata\core_imdb\imdb_t1ff_2015_f3_v1.dta", clear

keep IMDB_ID XTIRCF MKINCF EI___F TRPINF SASPYF EINS_F LIMXTI LIMATI NAIC*

save "T:\Projet 6899\Marwa_wokingfolder\Tax_files\2015.dta", replace

use "S:\IMDB_BDIM_AllYears\IMDB_BDIM_2020_v1\data_donnees\stata\core_imdb\imdb_t1ff_2016_f3_v1.dta", clear

keep IMDB_ID XTIRCF MKINCF EI___F TRPINF SASPYF EINS_F LIMXTI LIMATI NAIC*

save "T:\Projet 6899\Marwa_wokingfolder\Tax_files\2016.dta", replace

use "S:\IMDB_BDIM_AllYears\IMDB_BDIM_2020_v1\data_donnees\stata\core_imdb\imdb_t1ff_2017_f3_v1.dta", clear

keep IMDB_ID XTIRCF MKINCF EI___F TRPINF SASPYF EINS_F LIMXTI LIMATI NAIC*

save "T:\Projet 6899\Marwa_wokingfolder\Tax_files\2017.dta", replace

use "T:\Projet 6899\Marwa_wokingfolder\Tax_files\1999.dta", clear

merge 1:1 IMDB_ID using "T:\Projet 6899\Marwa_wokingfolder\Tax_files\2000.dta"

rename _merge m_2000

merge 1:1 IMDB_ID using "T:\Projet 6899\Marwa_wokingfolder\Tax_files\2001.dta"

rename _merge m_2001

merge 1:1 IMDB_ID using "T:\Projet 6899\Marwa_wokingfolder\Tax_files\2002.dta"

rename _merge m_2002

merge 1:1 IMDB_ID using "T:\Projet 6899\Marwa_wokingfolder\Tax_files\2003.dta"

rename _merge m_2003

merge 1:1 IMDB_ID using "T:\Projet 6899\Marwa_wokingfolder\Tax_files\2004.dta"

rename _merge m_2004

merge 1:1 IMDB_ID using "T:\Projet 6899\Marwa_wokingfolder\Tax_files\2005.dta"

rename _merge m_2005

merge 1:1 IMDB_ID using "T:\Projet 6899\Marwa_wokingfolder\Tax_files\2006.dta"

rename _merge m_2006

merge 1:1 IMDB_ID using "T:\Projet 6899\Marwa_wokingfolder\Tax_files\2007.dta"

rename _merge m_2007

merge 1:1 IMDB_ID using "T:\Projet 6899\Marwa_wokingfolder\Tax_files\2008.dta"

rename _merge m_2008

merge 1:1 IMDB_ID using "T:\Projet 6899\Marwa_wokingfolder\Tax_files\2009.dta"

rename _merge m_2009

merge 1:1 IMDB_ID using "T:\Projet 6899\Marwa_wokingfolder\Tax_files\2010.dta"

rename _merge m_2010

merge 1:1 IMDB_ID using "T:\Projet 6899\Marwa_wokingfolder\Tax_files\2011.dta"

rename _merge m_2011

merge 1:1 IMDB_ID using "T:\Projet 6899\Marwa_wokingfolder\Tax_files\2012.dta"

rename _merge m_2012

merge 1:1 IMDB_ID using "T:\Projet 6899\Marwa_wokingfolder\Tax_files\2013.dta"

rename _merge m_2013

merge 1:1 IMDB_ID using "T:\Projet 6899\Marwa_wokingfolder\Tax_files\2014.dta"

rename _merge m_2014

merge 1:1 IMDB_ID using "T:\Projet 6899\Marwa_wokingfolder\Tax_files\2015.dta"

rename _merge m_2015

merge 1:1 IMDB_ID using "T:\Projet 6899\Marwa_wokingfolder\Tax_files\2016.dta"

rename _merge m_2016

merge 1:1 IMDB_ID using "T:\Projet 6899\Marwa_wokingfolder\Tax_files\2017.dta"

rename _merge m_2017

save "T:\Projet 6899\Marwa_wokingfolder\Tax_files\1999_2017.dta", replace

*** Part 2 -subselection of study cohort from perinatal databse (X6) and meging into collated tax files by IMDBID- Please all the following quality check numbers should have changed by expansion of birth cohort

use "T:\Projet 6899\Working_Datasets_formatted\x6.dta", clear

keep if DOB_y >=2000 & DOB_y<=2017

save "T:\Projet 6899\Working_Datasets_formatted\refugee_cohort.dta", replace

** Next step is matching to tax files using IMDBID

merge m:1 IMDB_ID using "T:\Projet 6899\Marwa_wokingfolder\Tax_files\1999_2017.dta"

we need to drop those that only appear in tax documents but had no IMDBID (in using) to avoid confusion _merge == 2

*/

drop if _merge == 02

save "T:\Projet 6899\Marwa_wokingfolder\Tax_files\refugee_tax_2000_2017.dta" , replace

***Drop those with landing year prior to 1980

drop if LANDING_YEAR <1980

*******************************************************************************************************************************************

*** Part 3 - redefining some of the variables in the analytic dataset

***the following variables need to be redefined/ reconstructed before conducting the analysis to fillin descriptive tables

/*

1- receiving social assistance the year before birth

2- transit country

3- redefined immigration category

4- Income category

*/

*** generating a variable to understad proportion of women who filed taxes the year of birth

gen taxfile = .

replace taxfile = 0 if _merge == 03

forvalues i = 2000/2017{

gen taxfile`i'= `i' if XTIRCF`i' !=. & taxfile == 0

replace taxfile = 01 if taxfile == 0 & taxfile`i'== DOB_y

}

/*

*/

* First we are able to code for social assistance uisng a loop (coding has been modified to include soical assistance the year before delivery)

gen social_ass = .

replace social_ass = 0 if _merge == 03

forvalues i = 2000/2017{

gen assis`i'= `i' if SASPYF`i' >0 & social_ass == 0

replace social_ass = 01 if social_ass == 0 & assis`i'== DOB_y-1

}

gen sb_lb = .

replace sb_lb = 0 if LB_STC_ID !=""

replace sb_lb = 01 if SB_STC_ID !=""

save "T:\Projet 6899\Marwa_wokingfolder\Tax_files\refugee_tax_2000_2017.dta" , replace

*** 2-defining a varaible for transit country (This one needs to be redefined as it includes all birth cohort and we may want to limit it only to refugees)

use "T:\Projet 6899\Marwa_wokingfolder\Tax_files\refugee_tax_2000_2017.dta", clear

capture drop transit_cntry

gen transit_cntry=.

replace transit_cntry=0 if COUNTRY_BIRTH==COUNTRY_RESIDENCE

replace transit_cntry=1 if COUNTRY_BIRTH!=COUNTRY_RESIDENCE

label var transit_cntry "Lived in a transit country prior to immigrating to Canada"

**3- regrouping immigration category

** lets first understand existing variables- please note that the variable was constructed based on considering the nativity final of women

tab cb_immig_grps

*** construction of a new immigration Variable that includes

*Canadain Born- Economic- Family- GAR with dependents -PAR with dependents-Incanda refugees with dependents -other resettled refugees - others and one with nativity final issues

*** country_birth from original file to recategorize no 14

**some checks first

count if IMDB_ID !="" & foreign_born ==0

count if IMDB_ID =="" & foreign_born ==0

capture drop imm_gr_new

gen imm_gr_new = .

replace imm_gr_new = 0 if cb_immig_grps2==0

replace imm_gr_new = 01 if (cb_immig_grps2==1 | cb_immig_grps2==2 | cb_immig_grps2==3)

replace imm_gr_new = 02 if (cb_immig_grps2==4 | cb_immig_grps2==5 | cb_immig_grps2==6)

replace imm_gr_new = 03 if (cb_immig_grps2==7 | cb_immig_grps2==9) & IMMIGRATION_CATEGORY_CENSUS == "C3210" & (FAMILY_STATUS_ROLLUP == 1 | FAMILY_STATUS_ROLLUP==2)

replace imm_gr_new = 04 if (cb_immig_grps2==8 | cb_immig_grps2==9) & (IMMIGRATION_CATEGORY_CENSUS == "C3220" | IMMIGRATION_CATEGORY_CENSUS == "Z9991") & (FAMILY_STATUS_ROLLUP == 1 | FAMILY_STATUS_ROLLUP==2)

replace imm_gr_new = 05 if (cb_immig_grps2==11 | cb_immig_grps2==12)

replace imm_gr_new = 06 if cb_immig_grps2==10

replace imm_gr_new = 07 if cb_immig_grps2 ==13

replace imm_gr_new = 08 if cb_immig_grps2 ==14

*labeling values for clarity

capture drop imig_gr_new

label define imig_gr_new 0 "Canadian Born " 1 "Economic migrants " 2 "Family Class" 3 "GAR & dependents " 4 "PAR & dependents " 5 "In-Canada refugees & dependents " 6 "Other resettled refugees " 7 "others" 8 "other category-nativity final "

label values imm_gr_new imig_gr_new

tab imm_gr_new

*/

**The following may need to be considered at one point , seems nativity final is more accurate tan using mother birth registeration record

*** creating a new immigration category by foreign_born variable instead of nativity inal

gen imm_gr_new2 = imm_gr_new

replace imm_gr_new2 = 0 if foreign_born == 0

label values imm_gr_new2 imig_gr_new

tab imm_gr_new2 foreign_born

**** sub-dividing IN-CANADA refugees to those after landing nd those before landing

*** gen imm_gr_new3

capture drop imm_gr_new3

gen imm_gr_new3 = imm_gr_new

recode imm_gr_new3 (6=7)

recode imm_gr_new3 (7=8)

recode imm_gr_new3 (8=9)

recode imm_gr_new3 (5=5) if LANDING_YEAR <= DOB_y

recode imm_gr_new3 (5=6) if LANDING_YEAR > DOB_y

label define imig_gr_new3 0 "Canadian Born " 1 "Economic migrants " 2 "Family Class" 3 "GAR & dependents " 4 "PAR & dependents " 5 "Group A-In-Canada refugees & dependents " 6 "Group B-In-Canada refugees & dependents " 7 "Other resettled refugees " 8 "others" 9 "other category-nativity final "

** Group A delivery happened same year or after they were granted refugee status

** Group B delivery happened before they were granted refugee status

label values imm_gr_new3 imig_gr_new3

tab imm_gr_new3 imm_gr_new

*** DETERMINING the duration before granting refugee status

gen TIMETORYEAR= LANDING_YEAR-FIRST_EFFECTIVE_YEAR

bysort imm_gr_new3: sum TIMETORYEAR

*4- Income category

* generate income category as using the following variable

*XTIRCF-'Total income(ISD),family

gen income = .

replace income = XTIRCF2000 if DOB_y == 2000 & _merge == 03

replace income = XTIRCF2001 if DOB_y == 2001 & _merge == 03

replace income = XTIRCF2002 if DOB_y == 2002 & _merge == 03

replace income = XTIRCF2003 if DOB_y == 2003 & _merge == 03

replace income = XTIRCF2004 if DOB_y == 2004 & _merge == 03

replace income = XTIRCF2005 if DOB_y == 2005 & _merge == 03

replace income = XTIRCF2006 if DOB_y == 2006 & _merge == 03

replace income = XTIRCF2007 if DOB_y == 2007 & _merge == 03

replace income = XTIRCF2008 if DOB_y == 2008 & _merge == 03

replace income = XTIRCF2009 if DOB_y == 2009 & _merge == 03

replace income = XTIRCF2010 if DOB_y == 2010 & _merge == 03

replace income = XTIRCF2011 if DOB_y == 2011 & _merge == 03

replace income = XTIRCF2012 if DOB_y == 2012 & _merge == 03

replace income = XTIRCF2013 if DOB_y == 2013 & _merge == 03

replace income = XTIRCF2014 if DOB_y== 2014 & _merge == 03

replace income = XTIRCF2015 if DOB_y== 2015 & _merge == 03

replace income = XTIRCF2016 if DOB_y ==2016 & _merge == 03

replace income = XTIRCF2017 if DOB_y ==2017 & _merge == 03

gen income_cat =.

replace income_cat = 0 if income <35000 & income !=.

replace income_cat = 01 if income >=35000 & income < 60000

replace income_cat = 02 if income >=60000 & income < 90000

replace income_cat = 03 if income >= 90000 & income !=.

**** GENERATING ANOTHER INCOME VARIBLE FOR YEAR BEFORE DELIVERY

gen incybefore = .

replace incybefor = XTIRCF1999 if DOB_y == 2000 & _merge == 03

replace incybefor = XTIRCF2000 if DOB_y == 2001 & _merge == 03

replace incybefor = XTIRCF2001 if DOB_y == 2002 & _merge == 03

replace incybefor = XTIRCF2002 if DOB_y == 2003 & _merge == 03

replace incybefor = XTIRCF2003 if DOB_y == 2004 & _merge == 03

replace incybefor = XTIRCF2004 if DOB_y== 2005 & _merge == 03

replace incybefor = XTIRCF2005 if DOB_y== 2006 & _merge == 03

replace incybefor = XTIRCF2006 if DOB_y ==2007 & _merge == 03

replace incybefor = XTIRCF2007 if DOB_y ==2008 & _merge == 03

replace incybefor = XTIRCF2008 if DOB_y ==2009 & _merge == 03

replace incybefor = XTIRCF2009 if DOB_y == 2010 & _merge == 03

replace incybefor = XTIRCF2010 if DOB_y == 2011 & _merge == 03

replace incybefor = XTIRCF2011 if DOB_y == 2012 & _merge == 03

replace incybefor = XTIRCF2012 if DOB_y == 2013 & _merge == 03

replace incybefor = XTIRCF2013 if DOB_y == 2014 & _merge == 03

replace incybefor = XTIRCF2014 if DOB_y== 2015 & _merge == 03

replace incybefor = XTIRCF2015 if DOB_y== 2016 & _merge == 03

replace incybefor = XTIRCF2016 if DOB_y ==2017 & _merge == 03

gen income_dif = incybefore- income if _merge == 03

gen income_improv = .

replace income_improv = 0 if income_dif <=0 & income_dif !=.

replace income_improv = 01 if income_dif > 0 & income_dif !=.

tab income_improv

****update 20220408 we needed to adjust income to inflation factors using CPI

gen incybefore_ad = .

replace incybefore_ad = XTIRCF1999*1.382131324004 if DOB_y == 2000 & _merge == 03

replace incybefore_ad = XTIRCF2000*1.3459119 if DOB_y == 2001 & _merge == 03

replace incybefore_ad = XTIRCF2001*1.3128834 if DOB_y == 2002 & _merge == 03

replace incybefore_ad = XTIRCF2002*1.284 if DOB_y == 2003 & _merge == 03

replace incybefore_ad = XTIRCF2003*1.2490272 if DOB_y == 2004 & _merge == 03

replace incybefore_ad = XTIRCF2004*1.226361 if DOB_y== 2005 & _merge == 03

replace incybefore_ad = XTIRCF2005*1.2 if DOB_y== 2006 & _merge == 03

replace incybefore_ad = XTIRCF2006*1.1769019 if DOB_y ==2007 & _merge == 03

replace incybefore_ad = XTIRCF2007*1.1515695 if DOB_y ==2008 & _merge == 03

replace incybefore_ad = XTIRCF2008*1.1253287 if DOB_y ==2009 & _merge == 03

replace incybefore_ad = XTIRCF2009*1.1223776 if DOB_y == 2010 & _merge == 03

replace incybefore_ad = XTIRCF2010*1.1021459 if DOB_y == 2011 & _merge == 03

replace incybefore_ad = XTIRCF2011*1.0708924 if DOB_y == 2012 & _merge == 03

replace incybefore_ad = XTIRCF2012*1.0550534 if DOB_y == 2013 & _merge == 03

replace incybefore_ad = XTIRCF2013*1.0456026 if DOB_y == 2014 & _merge == 03

replace incybefore_ad = XTIRCF2014*1.0255591 if DOB_y== 2015 & _merge == 03

replace incybefore_ad = XTIRCF2015*1.014218 if DOB_y== 2016 & _merge == 03

replace incybefore_ad = XTIRCF2016 if DOB_y ==2017 & _merge == 03

***to add adjusted income cat

gen income_cat_ad =.

replace income_cat_ad = 0 if incybefore_ad <35000 & incybefore_ad !=.

replace income_cat_ad = 01 if incybefore_ad >=35000 & incybefore_ad< 60000

replace income_cat_ad = 02 if incybefore_ad >=60000 & incybefore_ad < 90000

replace income_cat_ad = 03 if incybefore_ad >= 90000 & incybefore_ad !=.

tab income_cat_ad

save "T:\Projet 6899\Marwa_wokingfolder\Tax_files\refugee_analytics.dta", replace

*** decision was made to drop immigration cateory labelled as canadian born with IMDB ID as well as other immigration categories

**1- Those with INCOME INFORMATION BUT CATEGORIES AS CANADAIN BORN (NO 11)

drop if imm_gr_new == 0 & income_cat !=.

*** generating avaraible for low income status - please note that this belongs to the year before delivery no information was found on adjustment by province in the code book

gen low_income = .

replace low_income = 0 if _merge == 03

forvalues i = 2000/2017{

gen loinc`i'= `i' if LIMATI`i' == "1"& low_income == 0

replace low_income = 01 if low_income == 0 & loinc`i'== DOB_y-1

}

/*

tab imm_gr_new low_income

*/

**we decided to drop those who belonged to others and nativity final

keep if imm_gr_new <7

*/

save "T:\Projet 6899\Marwa_wokingfolder\Tax_files\refugee_analytics.dta", replace

*** For descriptive analysis, some variables are missing from IMDP database like language and new syrian category

use "T:\Projet 6899\Working_Datasets_formatted\IMDB_FORMATTED\imdb_pnrf_1980_2018_f3_v1.dta", clear

keep IMDB_ID OFFICIAL_LANGUAGE SYRIAN_RRW

save "T:\Projet 6899\Marwa_wokingfolder\IMDB_language_syrian.dta", replace

use "T:\Projet 6899\Marwa_wokingfolder\Tax_files\refugee_analytics.dta", clear

rename _merge taxbirthm

merge m:1 IMDB_ID using "T:\Projet 6899\Marwa_wokingfolder\IMDB_language_syrian.dta"

drop if _merge ==02

/*

**/

save "T:\Projet 6899\Marwa_wokingfolder\Tax_files\refugee_analytics.dta", replace

use "T:\Projet 6899\Marwa_wokingfolder\Tax_files\refugee_analytics.dta", clear

*The following variables need to be categories for descriptive purposes

gen sex_cat= sex if sex !=09

tab sex_cat

gen language = OFFICIAL_LANGUAGE if OFFICIAL_LANGUAGE !=09

gen residence_cat = .

replace residence_cat = 0 if duration_of_residence <2 & duration_of_residence !=.

replace residence_cat = 01 if duration_of_residence >=2 & duration_of_residence< 6

replace residence_cat = 02 if duration_of_residence >=6 & duration_of_residence < 11

replace residence_cat = 03 if duration_of_residence >= 11 & duration_of_residence !=.

/*

*/

save "T:\Projet 6899\Marwa_wokingfolder\Tax_files\refugee_analytics.dta", replace

***************************************************************************

*** Part 4 - Data analysis

*** Before going into analysis, the decison was to clean the analytic datset from the following

use "T:\Projet 6899\Marwa_wokingfolder\Tax_files\refugee_analytics.dta", clear

*** first you need to limit the analysis to singleton pregnancies

tab plurality_d

drop if plurality_d == 2

save "T:\Projet 6899\Marwa_wokingfolder\Tax_files\refugee_analytics.dta", replace

**3- MOthers with multiple IMDBID (NO 22)

***taking a subset of mother and IMDB id to correct discrepancies \\

keep MOTHER_STC_ID IMDB_ID

gen check = .

collapse check, by (MOTHER_STC_ID IMDB_ID)

/*

duplicates report MOTHER_STC_ID

*** this indicates different mothers had same IMDBs

*/

/*

duplicates report IMDB_ID if IMDB_ID != ""

** this indicates that same mother had multiple IMDBs

*/

capture drop dup*

sort IMDB_ID

quietly by IMDB_ID: gen dup1 = cond(_N==01, 0, _n) if IMDB_ID != ""

/*

tab dup1

*/

sort MOTHER_STC_ID

quietly by MOTHER_STC_ID: gen dup2 = cond(_N==01, 0, _n)

/*

tab dup2

*/

save "T:\Projet 6899\Marwa_wokingfolder\Tax_files\DUPLICATE_imdb.dta", replace

/*

list if dup2 >= 1

*/

drop if dup2 >1

*** after identifying mothers with multiple IMDB_IDS , merging back with analytic database

save "T:\Projet 6899\Marwa_wokingfolder\Tax_files\DUPLICATE_imdb.dta", replace

use "T:\Projet 6899\Marwa_wokingfolder\Tax_files\refugee_analytics.dta", clear

rename _merge langsyrnmerge

merge m:1 MOTHER_STC_ID using "T:\Projet 6899\Marwa_wokingfolder\Tax_files\DUPLICATE_imdb.dta"

tab dup2

***cleaning inconsitencies : mothers who had multiple IMDBIDS (dup1) as well as different mothers with same IMDB ID (dup2)

drop if dup1 >=1 & dup1 !=.

drop if dup2 >=1

save "T:\Projet 6899\Marwa_wokingfolder\Tax_files\refugee_analytic_clean.dta", replace

/*

* from the above we conclude that the analytic cohort has a total of x live borths and still births , we made the following alterations to x6 DATASET

1- A cohort of 2000-2017 births was subselected

2- we dropped births of mothers with multiple IMDBIDS as well as births of mothers with same IMDB ID (see above)

3- We had issue with some mothers who were coded as canadian born however they had IMDBID so they were dropped

4- we dropped a group who whom immigrant stantus was coded as others or had issues with nativity final (see above)

5-we limited the analysis to singleton pregnancies

*/

use "T:\Projet 6899\Marwa_wokingfolder\Tax_files\refugee_analytic_clean.dta", clear

*** correcting the coding of some variables

encode WORLD_AREA_BIRTH, gen (world_region)

gen land_cohort = .

replace land_cohort = 0 if LANDING_YEAR<2 & LANDING_YEAR !=.

replace land_cohort = 01 if LANDING_YEAR >=1980 & LANDING_YEAR< 1990

replace land_cohort = 02 if LANDING_YEAR >=1990 & LANDING_YEAR< 2000

replace land_cohort = 03 if LANDING_YEAR >=2000 & LANDING_YEAR < 2010

replace land_cohort = 04 if LANDING_YEAR >=2010 & LANDING_YEAR <= 2018

*** check variable for educational status at landing - used same as Gabe

*** coding for a new education varaible

capture drop education_clean

gen education_clean = .

replace education_clean = 0 if education_IMDB_c == 01 | education_IMDB_c ==2

replace education_clean = 1 if education_IMDB_c == 3

replace education_clean = 2 if education_IMDB_c == 4

replace education_clean = 3 if education_IMDB_c == 5

label define educationclean 0 " less than High School" 1 "High school diploma" 2 "Bachelor's" 3 "Master or PhD"

label values education_clean educationclean

tab education_clean

save "T:\Projet 6899\Marwa_wokingfolder\Tax_files\refugee_analytic_clean.dta", replace

*outcome rates with CIs by year of birth for each immigrant group

*tab1 ptb sga lga stillbirth death sb_500_20 sb_25 DOB_y DOB_y_g

*tab imm_gr_new neonatal

*tab imm_gr_new post_neonatal

capture log close

use "T:\Projet 6899\Marwa_wokingfolder\Tax_files\refugee_analytic_clean.dta", clear

log using "T:\Projet 6899\Marwa_wokingfolder\refugee_results_.log", replace

keep if imm_gr_new3 < 07 & imm_gr_new3 !=2

tab imm_gr_new3

**Table 1 descriptive statistics - sociodemographic

foreach y of varlist parity_3cat sex_cat m_age_c marital_c region world_region transit_cntry land_cohort residence_cat education_clean income_cat_ad language social_ass low_income{

table (`y') imm_gr_new3

proportion `y', over(imm_gr_new3)

}

*** Table 2 -perinatal outcomes - all groups except family class

*** Descriptve for all migrants groups including canadian born

foreach y of varlist ptb sga lga stillbirth death neonatal post_neonatal sb_500_20 sb_25 {

table (`y') imm_gr_new3

proportion `y', over(imm_gr_new3)

}

** the following log file is for generating different P values if needed for Tables 1-2

capture log close

log using "T:\Projet 6899\Marwa_wokingfolder\refugee_results_20220414_extended.log", replace

foreach y of varlist parity_3cat sex_cat DOB_y m_age_c marital_c_rev region WR12 land_cohort residence_cat_rev age_m_r educ_clean_rev language social_ass {

tab (`y') imm_gr_new3, chi

}

foreach y of varlist ptb sga lga stillbirth death neonatal post_neonatal sb_500_20 sb_25 {

tab (`y') imm_gr_new3, chi

}

foreach y of varlist ptb PTB_3 sga lga stillbirth death neonatal post_neonatal sb_500_20 sb_25 {

tab (`y') transit_cntry if imm_gr_new3 == 3, chi

tab (`y') transit_cntry if imm_gr_new3 == 4, chi

tab (`y') transit_cntry if imm_gr_new3 == 5, chi

tab (`y') transit_cntry if imm_gr_new3 == 6, chi

}

log close

***Part 4-b Analystic statiscs

**Tables 3 and 4 -

*use "T:\Projet 6899\Marwa_wokingfolder\Tax_files\refugee_analytic_clean.dta", clear

keep if imm_gr_new3 < 07 & imm_gr_new3 !=2 & imm_gr_new3 !=0

foreach y of varlist PTB SGA LGA stillbirth death sb_500_20 sb_25 {

xtgee `y' i.imm_gr_new3, family(binomial) link(log) corr(exc) robust i(mother_id)

margins r.imm_gr_new3

xtgee `y' i.imm_gr_new3 i.parity_3cat sex_cat i.DOB_y i.m_age_c i.marital_c_rev i.region ///

, family(binomial) link(log) corr(exc) robust i(mother_id)

margins r.imm_gr_new3

xtgee `y' i.imm_gr_new3 i.parity_3cat sex_cat i.DOB_y i.m_age_c i.marital_c_rev i.region i.WR12 i.land_cohort i.age_m_r ///

, family(binomial) link(log) corr(exc) robust i(mother_id)

margins r.imm_gr_new3

xtgee `y' i.imm_gr_new3 i.parity_3cat sex_cat i.DOB_y i.m_age_c i.marital_c_rev i.region i.WR12 i.land_cohort i.residence_cat_rev i.age_m_r i.educ_clean_rev i.language social_ass ///

, family(binomial) link(log) corr(exc) robust i(mother_id)

margins r.imm_gr_new3

}

*** excluding economic migrants

keep if imm_gr_new !=01

foreach y of varlist PTB SGA LGA stillbirth death sb_500_20 sb_25 {

xtgee `y' i.imm_gr_new3, family(binomial) link(log) corr(exc) robust i(mother_id)

margins r.imm_gr_new3

xtgee `y' i.imm_gr_new3 i.parity_3cat sex_cat i.DOB_y i.m_age_c i.marital_c_rev i.region ///

, family(binomial) link(log) corr(exc) robust i(mother_id)

margins r.imm_gr_new3

xtgee `y' i.imm_gr_new3 i.parity_3cat sex_cat i.DOB_y i.m_age_c i.marital_c_rev i.region i.WR12 i.land_cohort i.age_m_r ///

, family(binomial) link(log) corr(exc) robust i(mother_id)

margins r.imm_gr_new3

xtgee `y' i.imm_gr_new3 i.parity_3cat sex_cat i.DOB_y i.m_age_c i.marital_c_rev i.region i.WR12 i.land_cohort i.residence_cat_rev i.age_m_r i.educ_clean_rev i.language social_ass ///

, family(binomial) link(log) corr(exc) robust i(mother_id)

margins r.imm_gr_new3

}
